# Leveraging data science and AI to democratize global surgical expertise

**DOI:** 10.1136/bmjsit-2024-000334

**Published:** 2024-11-29

**Authors:** Samy Cheikh Youssef, Prokar Dasgupta, May Haram, Nadine Hachach-Haram

**Affiliations:** 1 St George's University of London, London, UK; 2 King's College London, London, UK; 3 King's Health Partners, London, UK; 4 Godolphin and Latymer School, London, UK; 5 Plastic Surgery, Guy's and Saint Thomas' NHS Foundation Trust, London, UK

**Keywords:** Robotic Surgical Procedures, Health Technology, Technology

## Introduction

 By 2025, the volume of healthcare data is expected to exceed 10 trillion gigabytes.^1^ Data science has already permeated many facets of healthcare, from the automated interpretation of medical imaging to the timely prediction of postoperative complications. The intersection of data science and artificial intelligence (AI) represents a paradigm shift in healthcare.^2^ Rooted in the early developments of medical informatics and computer-assisted surgery, surgical data science has evolved into a broad discipline leveraging diverse forms of data.

From the creation of the first electronic health record (EHR) and the shift to computerized documentation in the 1960s to the data-driven AI applications witnessed in healthcare today, the growing libraries of patient data necessitate the appropriate infrastructure for processing and utilization. This article explores the integral role of data interoperability in the development of this infrastructure, and how it can facilitate the development of AI, bridging gaps in surgical practice, academia and democratizing surgical care globally.

### Current landscape of surgical data science and the role of interoperability

The current operating room is characterized by an immense influx of data, generated from increasingly diverse sources, as medical technology advances. Beyond the sophisticated kinematic data from robotic surgical systems, before surgery begins—written data in patient EHRs can offer vital clues to a patient’s intraoperative journey and postoperative recovery. Intraoperatively, devices monitor patient vital signs, surgical robots collect metrics on the console surgeon, and theatre coordinators record key time stamps. Postoperatively, recovery is closely monitored and documented in EHRs. In some institutions, video and audio recordings are routinely collected providing a rich, yet complex source of data.^3^ Additionally, visual, spatial, and temporal annotations may be applied to enrich the data for various purposes; from the production of educational materials to the training of machine learning algorithms.

Understanding the challenges in using surgical data, makes evident the obstacles in training AI algorithms for the surgical context. The variability in the data collected, methods of collection, storage, organization, and analysis are what lead to a fragmented data ecosystem.

A 2019 Delphi study by Maier-Hein *et al* and leading researchers in surgical data science, highlighted the need for standardized technical infrastructure to enable the acquisition, storage, and access to data in surgical practice.^4^ The study emphasized that interoperable platforms are essential for streamlined data collection and utilization downstream. Despite recent progress, a survey of nine video recording technology companies revealed significant variability in storage methods, metadata application, and AI features for surgical video.^5^ In 2023, a further Delphi study proposed guidelines for standardizing the recording and processing of surgical video data, the purpose of which would enhance its utility for both clinical and non-clinical stakeholders and facilitate cross-institutional data exchange.^6^

### How surgical data science can transform surgical practice

AI-driven systems benefit not only surgical personnel, but also the broader healthcare ecosystem. For management, AI optimizes operational efficiency by predicting theatre time and improving scheduling.^7^ Being able to predict remaining time based on OR context, has both clinical and non-clinical benefits. Clinically, identifying aberrations from the expected workflow enables necessary interventions from staff outside the OR pre-emptively, as a centralized theatre progress panel shows the OR status. For non-clinical stakeholders, being able to more accurately predict and track theater time—minimizes turnover time improving efficiency.

Identifying institution-specific factors that influence metrics like turnover time, may also enable targeted interventions, ultimately improving resource allocation. The AI application described is termed “descriptive” by Maier-Hein *et al*, with an indirect role in the surgical workflow as opposed to prescriptive applications—which have an intraoperative role. These applications pose a lower barrier of entry with hospital stakeholders, while still showcasing the potential AI can provide.^4^

**Figure 1:**
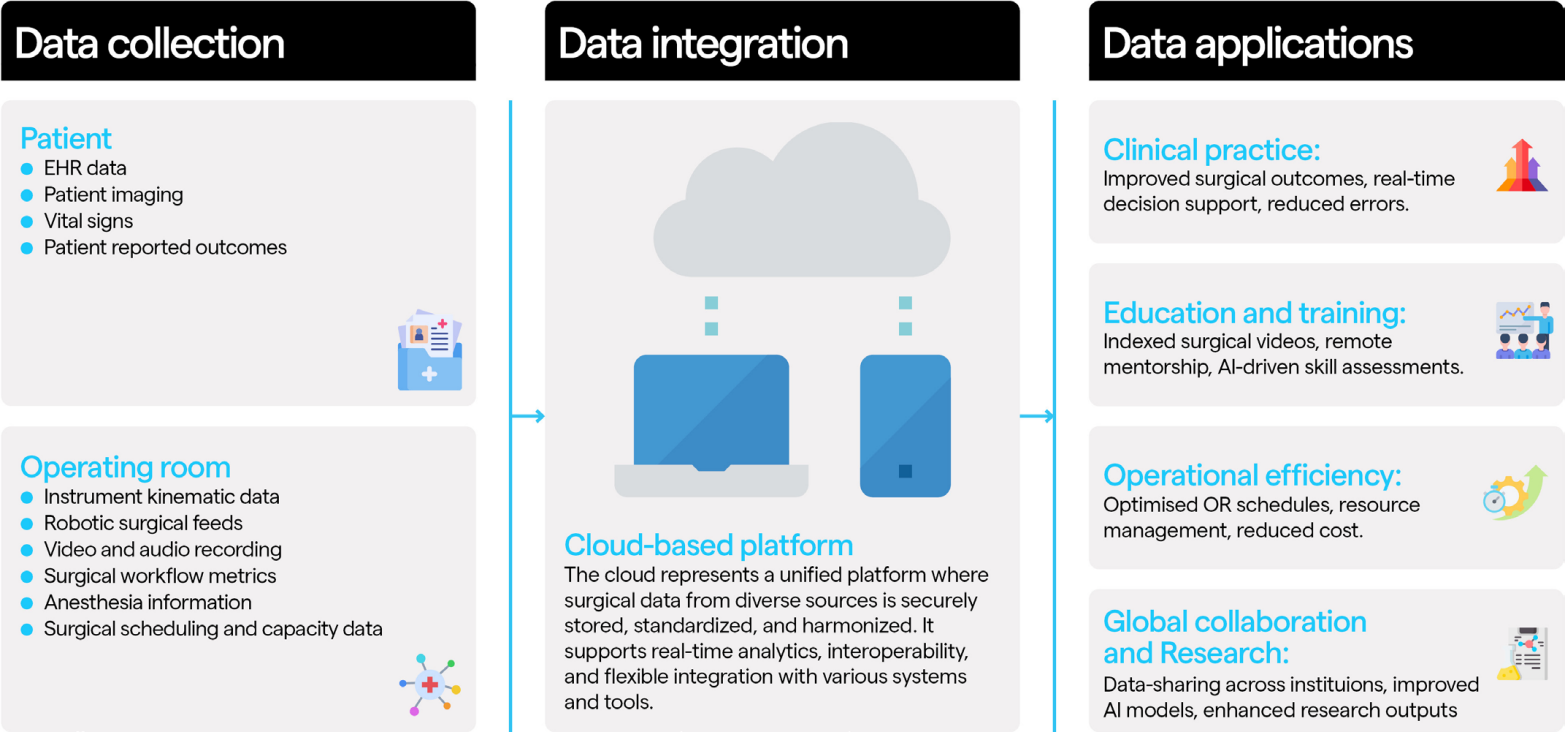
Data **flow for training surgical** AI applications. This figure demonstrates the flow of data, where it originates and the final applications when unified, and carefully curated for the purposes of training AI algorithms. Data Collection is divided into two primary sources: patient-related data (eg, EHR records, vital signs, imaging) and OR-related data, sourced from various inputs depending on the specific setup and culture of the OR (eg, video recordings, robotic surgery feeds, kinematic data). Data Integration is when the wealth of data from these sources is unified on a centralized platform, organized, and standardized in format. This enables ease of accessibility for authorized stakeholders. Data Applications, on the right of the figure showcases some of the existent applications of AI algorithms and surgical data science to date. As infrastructure develops, and the field advances innovation is likely to broaden the range of uses. AI, artificial intelligence; EHR, electronic health record.

For surgeons and trainees, AI algorithms can be trained to provide intraoperative insights on patient anatomy and risk prediction, facilitating safer surgery and subsequently improved patient outcomes.^8^ Postoperatively, indexed videos facilitate reflective practice, mentoring, and teaching, enhancing technical skills and clinical decision-making. For surgical academics, access to multimodal data offers opportunities for research initiatives, which, if properly considered, could retrospectively provide data that inspires innovation in current surgical practice (figure 1).^9^

### Challenges and considerations

While the integration of AI and data science into surgery offers substantial benefits, it also presents challenges. Data points collected during the surgical workflow often exist in isolation, representing only fragments of the patient pathway. Identifying all the relevant variables and routinely collecting them, requires investment in infrastructure, training, and multidisciplinary collaboration. Such requirements can be prohibitive in low-resource settings. However, ensuring interoperability between diverse health systems and adopting data collection across different regions is critical to mitigating bias and training the most capable AI models.^6^

Complex ethical concerns surrounding patient data privacy and regulatory bodies such as GDPR and HIPAA in the UK and USA, could hinder the development of globally applicable AI models. Moreover, as of August 1, 2024, the first formal legislation, the “AI Act,” was announced, detailing a comprehensive legal framework identifying high-risk applications. Of which, the use of AI in healthcare, and robot-assisted surgery specifically may face obstacles and scrutiny. Navigating such guidelines—particularly for algorithms using patients’ biometric data or a “Black box” design—may face significant barriers, stifling implementation in healthcare.^10^

Robust frameworks are imperative to protect patient confidentiality while also advancing the field. Addressing these challenges requires a multifaceted approach, engaging numerous stakeholders. The provision of guidance by governmental policymakers, and medical technology companies’ careful consideration of guidelines and ongoing vigilance, are quintessential to develop an ethical and standardized approach to sensitive healthcare data.

## Conclusion

Future advancements in AI and surgical data science will demand considerable investment in enhancing data interoperability. The goal is to develop infrastructure capable of standardizing and unifying data from various sources, despite the diversity in infrastructure of each OR, while addressing regulatory and ethical challenges. Developing this will not only allow hospitals to benefit from their own data, but also facilitate the contribution to the global surgical community, leveraging the wealth of knowledge available, across institutions and borders.

## References

[1] Burgener E, Goodwin P, Piai S (2021). Establishing uncompromising data availability for healthcare organizations.

[2] Hashimoto DA, Rosman G, Rus D (2018). Artificial Intelligence in Surgery: Promises and Perils. Ann Surg.

[3] Yiu A, Lam K, Simister C (2024). Adoption of routine surgical video recording: a nationwide freedom of information act request across England and Wales. EClinMed.

[4] Maier-Hein L, Eisenmann M, Sarikaya D (2022). Surgical data science – from concepts toward clinical translation. Med Image Anal.

[5] Filicori F, Bitner DP, Fuchs HF (2023). SAGES video acquisition framework-analysis of available OR recording technologies by the SAGES AI task force. Surg Endosc.

[6] Eckhoff JA, Rosman G, Altieri MS (2023). SAGES consensus recommendations on surgical video data use, structure, and exploration (for research in artificial intelligence, clinical quality improvement, and surgical education). Surg Endosc.

[7] Bellini V, Russo M, Domenichetti T (2024). Artificial Intelligence in Operating Room Management. J Med Syst.

[8] Mascagni P, Alapatt D, Sestini L (2022). Computer vision in surgery: from potential to clinical value. NPJ Digit Med.

[9] Maier-Hein L, Vedula S, Speidel S Surgical data science: enabling next-generation surgery.

[10] European Commission DS (2024). AI act | shaping europe’s digital future. https://digital-strategy.ec.europa.eu/en/policies/regulatory-framework-ai.

